# Genome Scan of *M. tuberculosis* Infection and Disease in Ugandans

**DOI:** 10.1371/journal.pone.0004094

**Published:** 2008-12-31

**Authors:** Catherine M. Stein, Sarah Zalwango, LaShaunda L. Malone, Sungho Won, Harriet Mayanja-Kizza, Roy D. Mugerwa, Dmitry V. Leontiev, Cheryl L. Thompson, Kevin C. Cartier, Robert C. Elston, Sudha K. Iyengar, W. Henry Boom, Christopher C. Whalen

**Affiliations:** 1 Department of Epidemiology and Biostatistics, Case Western Reserve University, Cleveland, Ohio, United States of America; 2 Tuberculosis Research Unit, Department of Medicine, Case Western Reserve University, Cleveland, Ohio, United States of America; 3 Department of Family Medicine, Case Western Reserve University, Cleveland, Ohio, United States of America; 4 Uganda – CWRU Research Collaboration, Kampala, Uganda; 5 Makerere University School of Medicine and Mulago Hospital, Kampala, Uganda; McGill University, Canada

## Abstract

Tuberculosis (TB), caused by *Mycobacterium tuberculosis* (Mtb), is an enduring public health problem globally, particularly in sub-Saharan Africa. Several studies have suggested a role for host genetic susceptibility in increased risk for TB but results across studies have been equivocal. As part of a household contact study of Mtb infection and disease in Kampala, Uganda, we have taken a unique approach to the study of genetic susceptibility to TB, by studying three phenotypes. First, we analyzed culture confirmed TB disease compared to latent Mtb infection (LTBI) or lack of Mtb infection. Second, we analyzed resistance to Mtb infection in the face of continuous exposure, defined by a persistently negative tuberculin skin test (PTST-); this outcome was contrasted to LTBI. Third, we analyzed an intermediate phenotype, tumor necrosis factor-alpha (TNFα) expression in response to soluble Mtb ligands enriched with molecules secreted from Mtb (culture filtrate). We conducted a full microsatellite genome scan, using genotypes generated by the Center for Medical Genetics at Marshfield. Multipoint model-free linkage analysis was conducted using an extension of the Haseman-Elston regression model that includes half sibling pairs, and HIV status was included as a covariate in the model. The analysis included 803 individuals from 193 pedigrees, comprising 258 full sibling pairs and 175 half sibling pairs. Suggestive linkage (p<10^−3^) was observed on chromosomes 2q21-2q24 and 5p13-5q22 for PTST-, and on chromosome 7p22-7p21 for TB; these findings for PTST- are novel and the chromosome 7 region contains the *IL6* gene. In addition, we replicated recent linkage findings on chromosome 20q13 for TB (p = 0.002). We also observed linkage at the nominal α = 0.05 threshold to a number of promising candidate genes, *SLC11A1* (PTST- p = 0.02), IL-1 complex (TB p = 0.01), *IL12BR2* (TNFα p = 0.006), *IL12A* (TB p = 0.02) and *IFNGR2* (TNFα p = 0.002). These results confirm not only that genetic factors influence the interaction between humans and Mtb but more importantly that they differ according to the outcome of that interaction: exposure but no infection, infection without progression to disease, or progression of infection to disease. Many of the genetic factors for each of these stages are part of the innate immune system.

## Introduction

Tuberculosis (TB), caused by the bacterium *Mycobacterium tuberculosis* (Mtb), is a significant, global public health problem, particularly in sub-Saharan Africa, where the prevalence of TB is increasing dramatically with the rise of the HIV pandemic. One-third of the world is infected by Mtb [1]. According to the World Health Organization, almost 8 million new cases of TB occur annually, with 2 million deaths attributed to the disease each year. Uganda is one of the world's 22 highest burden countries with TB, with an estimated annual risk of tuberculosis infection of 3% and an annual incidence of new smear positive TB cases of 9.2 per 1000 in an urban setting [2]. An increased understanding of the host response to Mtb will facilitate the development of new vaccines and therapeutics [3].

Since only 10% of individuals infected with Mtb go on to develop active disease (TB), it has been suggested that host genetics may influence the risk for TB. Early evidence for susceptibility for TB was suggested by twin studies and difference in susceptibility observed among different human populations. Numerous candidate gene studies have been conducted for TB; though there is consistent support for a role of human genetics in disease risk, the results for specific genes have been equivocal (reviewed in [4]). Four genome-wide linkage scans have been conducted [5]–[8], but their results did not replicate each other. This may be due to differences in TB diagnostic criteria, population genetic differences (Brazilian, Gambian, Malawian and South African), small sample size, or analytic approach.

The pathogenesis of TB can be thought of as a two-stage process [9]. The first stage consists of latent Mtb infection (LTBI), in which Mtb establishes a productive infection but does not produce symptoms. LTBI is diagnosed by a positive tuberculin skin test (TST) and/or positive interferon-γ response assay (IGRA) and the absence of clinical signs and symptoms of full-blown disease [10]. Interestingly, some people remain uninfected, evidenced by a negative TST and/or negative IGRA, despite prolonged exposure to an infectious TB case. Negative TSTs occur even in TB-endemic settings, where exposure to Mtb is known to be persistent [10]; repeated negative TSTs over several occasions are even less likely to be false-negatives. These individuals are thought to be resistant to Mtb infection, but have not been studied extensively because few studies assess exposure to an infectious TB case within a home and prospectively conduct repeat TSTs. Previous studies have shown ethnic differences in the prevalence of LTBI and the rate of TST conversion [11] and in the relative permissiveness of macrophages to Mtb infection [12]. Another study showed differences in *FOXP3* gene expression between TST− and TST+ individuals [13]; however, it was unknown if those TST− individuals ever converted their TST, which is possible since they were contacts of TB cases, so they cannot be definitively classified as resistant to Mtb infection. These findings have suggested a role for human genetics in resistance to Mtb infection [14], but this has not been examined in a formal genetic epidemiological study. The second stage is TB (disease), in which Mtb replication and the host immune response disrupts normal physiology and produces characteristic signs and symptoms including productive cough and cavities on chest x-ray. It is this stage, development of TB, which has been studied in previous genetic epidemiological studies as well as mouse models.

In our previous studies [15]–[17], we have taken a unique approach to the study of TB genetics by studying tumor necrosis factor-α (TNFα) expression as an intermediate phenotype. Intermediate phenotypes are more closely tied to underlying disease biology [18], [19]. We have focused on TNFα because it is a central cytokine in TB pathogenesis that is involved in granuloma formation, induces symptoms including fever and weight loss [20], [21], and is important in the containment of latent Mtb infection [22]. Our previous studies have suggested that TNFα responses to Mtb are strongly influenced by genetic factors [15], [17], and that genes in the TNFα pathway also influence TB [16]. Genome-wide linkage studies have examined genetic influences on host immunity, and include studies of immune response to Mtb [23], IgE levels in asthma [24], and CD4+ and CD8+ cell levels in healthy persons [25].

Here we present a whole genome scan conducted in a household contact study of Mtb infection and disease in Kampala, Uganda. We focused on three phenotypes: persons with TB, compared to persistent TST− persons and TST+ LTBI individuals in their households; resistance to Mtb infection, compared to persons with LTBI; and TNFα as a continuous intermediate phenotype, measured on all study subjects. Analysis of these three phenotypes allowed us to examine the clinical spectrum from Mtb exposure to pulmonary TB. We hypothesized that these three distinct phenotypes each would have have unique genetic influences. This is the first genome scan to be conducted in Uganda, the largest linkage scan for TB, and the first to study resistance to Mtb infection.

## Results

**Figure 1:**
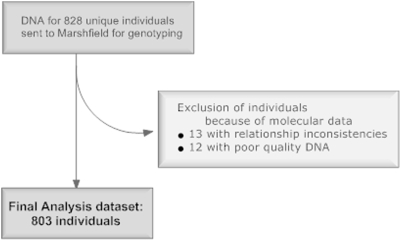
Flow diagram showing study sample before and after data cleaning.

After data cleaning (Figure 1), we analyzed a total of 193 pedigrees comprising 803 individuals (Table 1). Of these, 95 households were enrolled in Phase I of the study and 98 households were enrolled in Phase II. Both children and adults were included in the analysis. A total of 160 individuals had culture confirmed TB, 130 were HIV infected, and 85 were PTST-. Most of the PTST- and LTBI individuals were not HIV infected (89.6% and 91%, respectively); slightly more than half (51.9%) of the TB patients were HIV infected, which is consistent with our previous study in this population [26] (Table 2). HIV tests were not conducted for 66 very young children, since they were not at risk for HIV infection because neither parent was HIV infected. For linkage analysis, there were 258 full sibling pairs and 175 half sibling pairs.

**Table 1:** Descriptive statistics for final analysis sample.

Total number of individuals	803
Numbers of females/males	435/368
Median age (range)	15.00 (1–80)
Number of individuals with TB	160
Number of PTST- individuals	85
Number of HIV infected individuals	130
Number of households	193
Median pedigree size (range)	4.00 (2–24)
Number of full sibling pairs	258
Number of half sibling pairs	175

**Table 2:** Clinical group by HIV status.

	HIV negative	HIV positive
**PTST-**	69	8
**LTBI**	455	45
**TB**	83	77

HIV tests were not conducted for 66 children because they were not clinically relevant.

**Figure 2:**
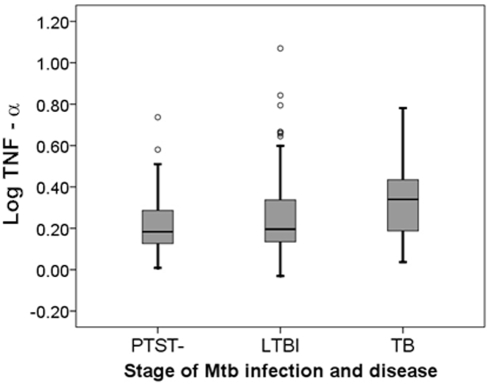
Boxplot of covariate-adjusted and log-transformed TNFα across stage of Mtb infection and disease. This boxplot displays the median and distribution of TNFα values across the three clinical groups. PTST- = persistent TST negative (resistant to Mtb infection), LTBI = latent Mtb infection (TST positive, no disease), TB = tuberculosis (disease).

We compared TNFα levels in the three clinical groups (PTST-, LTBI, and TB) after covariate adjustment and log-transformation. Boxplots illustrating the median and distribution of these adjusted and transformed TNFα values are provided in Figure 2. TNFα levels differed significantly between TB and both PTST- (p = 0.009) and LTBI (p = 0.009) in HIV negative individuals. TNFα levels did not differ significantly between PTST- and LTBI (p = 0.389). This analysis further illustrates that TNFα expression varies with TB progression.

Linkage analysis results are summarized in Table 3, which lists regions attaining significance at the nominal α = 0.05 level. In addition, the information content in those regions, as well as the candidate genes in the vicinity of these regions, is also listed. Linkage findings for TNFα levels are provided in two ways: in the first column, pooled results are provided, and in the second column, results for Phase II of the study analyzed alone are provided, since previous results suggested these TNFα phenotypes were different [15].

**Table 3:** Chromosomal regions significant at the α = 0.05 level by trait.

Chromosomal region	cM range^a^	Marker information (range)	Most significant p-value by trait				Genes in vicinity/Notes
			TB	PTST-	TNFα (meta)^b^	TNFα (Phase II)^b^	
1p31	120–130	.72–.80			0.014	0.006	15 Mb from *IL12RB2*
1p21-1q24	130–180	.71–.80	0.02				
2p22-2p16	54–80	.68–.80				0.02	
2p13-2q11	86–108	.72–.81	0.02				*IL1* complex
2q14	120–128	.75–.76		0.007			
2q21-2q24	146–176	.68–.75		**0.0003**			
2q27	250–260	.73–.80		0.02			17 Mb from *SLC11A1*
3q23	150–168	.73–.75	0.02				*IL12A*
5p13-5q22	64–114	.69–.77		**0.0005**			
6p21	36–50	.76–.86	0.03				*TNF/MHC*
7p22-7p21	0–34	.68–.82	**0.0002**				*IL6*
7q35-7q36	158–172	.51–.57		0.02			
8p22	32–40	.57–.73		0.04			Linked in Morrocan study[30]
8p12-8q11	64–72	.73–.77	0.001	0.02		0.02	
8q21-8q23	104–124	.64–.75			0.010	0.02	
9p21-9q12	54–66	.72–.78				0.02	
10q24-10q24	121–163	.57–.81	0.02				
11p15	0–16	.69–.73	0.02				
11q14-11q23	88–118	.72–.84			0.034	0.007	
14p13-14q11	2–24	.51–.77		0.02			
14q21-14q24	55–82	.64–.83		0.02			
19p13-19q12	40–54	.75–.76				0.006	
20q13	64–89	.47–.65	**0.002**				*MC3R* and *CTSZ*
21q22	38–44	.56–.62			0.009	0.01	*IFNGR2*
22p13-22q11	2–46	.65–.76	0.02			0.02	

cM = centimorgans, Mb = Megabases.

P-values in boldface indicate results attaining suggestive significance according to Lander-Kruglyak criteria.

a cM range indicates locations significant at the nominal α = 0.05 level.

b The first column of linkage results for the TNFα phenotype are for pooled p-values, and the second column is for the Phase II data analyzed alone.

**Figure 3:**
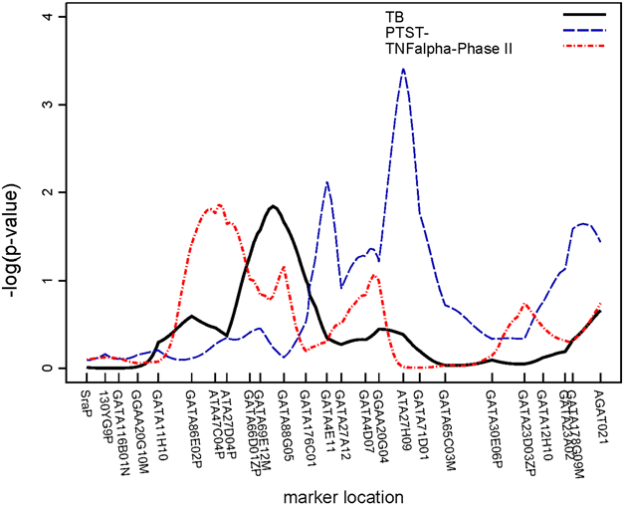
Plot of linkage results for chromosome 2. −log_10_(p-value) from the linkage analysis of each trait is plotted against marker location. This plot illustrates that different TB phenotypes were linked to different regions on chromosome 2.

**Figure 4:**
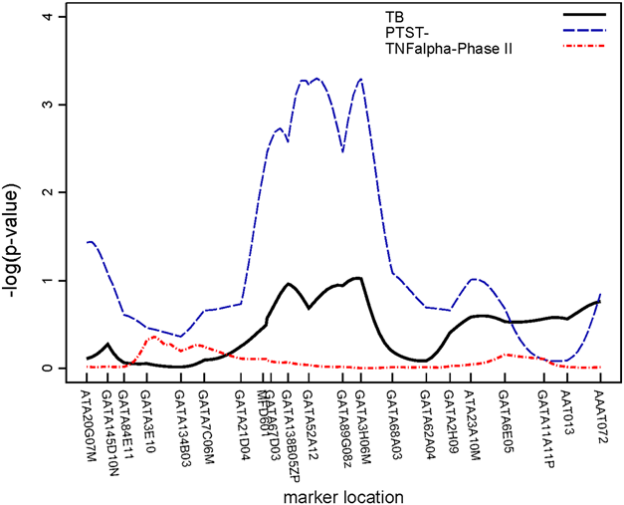
Plot of linkage results for chromosome 5. −log_10_(p-value) from the linkage analysis of each trait is plotted against marker location.

**Figure 5:**
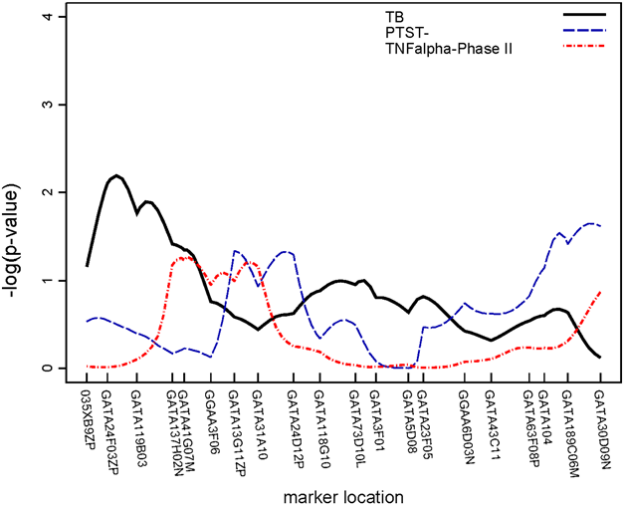
Plot of linkage results for chromosome 7. −log_10_(p-value) from the linkage analysis of each trait is plotted against marker location.

**Figure 6:**
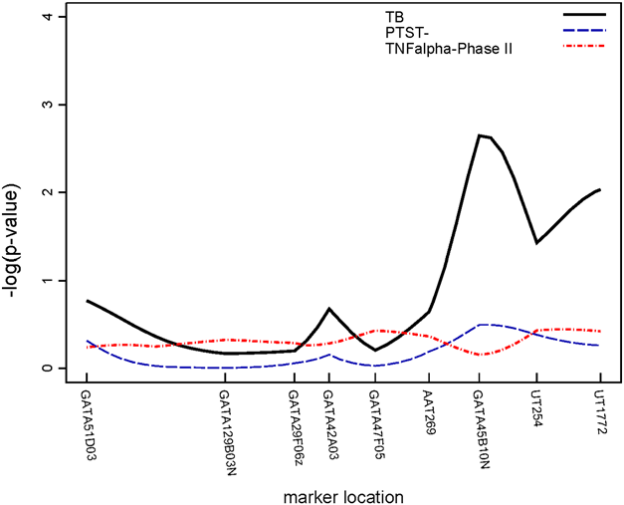
Plot of linkage results for chromosome 20. −log_10_(p-value) from the linkage analysis of each trait is plotted against marker location.

Though none of the regions attained genome-wide significance by conventional standards (p<10^−5^) [27], three regions attained suggestive significance (p<10^−3^). Two of these regions were linked to PTST-, one on chromosome 2 (p = 0.0003) (Figure 3) and another on chromosome 5 (p = 0.0005) (Figure 4). The region on chromosome 2q21-2q24 is 30 cM long and the region on chromosome 5p13-5q22 is 50 cM long; neither region contains any previously characterized candidate genes. A 34 cM long region on chromosome 7, linked to TB, also attained suggestive linkage (p = 0.0002) (Figure 5); the interleukin (IL)-6 gene resides at the centromeric end of this region. Though not quite attaining the suggestive significance threshold, we also observed linkage to TB on chromosome 20q13 (p = 0.002) (Figure 6). This same region, which was 25 cM long in this analysis, has recently been mapped in African populations [8] and found to contain genes for melanocortin 3 (*MC3R*) and cathepsin 7 (*CTSZ*). There were no regions attaining genome-wide significance or suggestive significance for TNFα.

In addition, a number of regions were linked at the α = 0.05 level to chromosomal regions in which possible TB candidate genes reside. Since linkage effects may extend up to 20 cM [28], we also considered linkage results within 20 cM of previously characterized candidate genes as possible replications. TNFα level, as assayed in both study phases, was linked to chromosome 1 (total sample p = 0.014, Phase II p = 0.006); the linked region (120–130 cM) is roughly 15 Mb from the interleukin (IL)-12 receptor β2 (*IL12RB2*). Another region on chromosome 1, extending from 130 through 180 cM, was linked to TB (p = 0.01). On chromosome 2 (Figure 3), a region extending from 250 to 260 cM was also linked to PTST- (p = 0.02); this region is approximately 17 Mb from the location of *NRAMP1 (SLC11A1)*, the most analyzed candidate gene in TB [29]. A second region on chromosome 2, between 86–108 cM, was linked to TB (p = 0.01); this region contains genes for the IL-1 complex (*IL1*, *IL1RA*, etc.). Another region on chromosome 2, extending from 54–80 cM, was linked to TNFα level in the Phase II sample (p = 0.02) but, because this region ends only 6 cM from the region linked to TB, it cannot be distinguished from the IL1 complex region. Other suggestive linkages to TB alone were seen on: chromosome 3, in a region containing *IL12A* (p = 0.02); chromosome 6, in the region containing the MHC complex and *TNFA* (p = 0.03); and the chromosome 7 and 20 regions mentioned above. Finally, a region on chromosome 21, in which the gene for IFNγ receptor 2 (*IFNGR2*) resides, was linked to TNFα levels from the whole sample (p = 0.009) and TNFα from Phase II (p = 0.01).

Several other regions attained linkage at the α = 0.05 level, but did not contain any previously characterized TB candidate genes. One region on chromosome 2, extending from 120–128 cM, was linked to PTST- (p = 0.007), and a second region ∼18 cM away, extending from 146–176 cM, was also linked to PTST- (p = 0.0003). Markers on the short arm of chromosome 8 were linked to all three phenotypes in the region between 32 and 72 cM, though only TNFα levels from the whole sample attained significance with p<0.01. Linkage to this chromosome 8 region has been previously reported in a Moroccan study population [30], but the specific gene(s) in this region have not been characterized.

To explore whether these results were confounded by the presence of HIV positive individuals in the analysis, we repeated the genome scan by restricting the analysis to sibling pairs that were concordantly HIV negative (Table 4). In general, most of the significant results remained, though some candidate regions were no longer significant or less significant in this restricted analysis. A few regions actually increased in their statistical significance. It is likely that the fluctuations in p-values were due to reductions in sample size for these analyses of subpopulations. Finally, we note that the information content, which is a measure that depends on the informativity of both the markers and the trait being analyzed, is 0.72.

**Table 4:** Chromosomal regions significant at the α = 0.05 level by trait for analysis of HIV concordantly negative sibling pairs.

Chromosomal region	cM range^a^	Marker information (range)	Most significant p-value by trait				Genes in vicinity/Notes
			TB	PTST-	TNFα (meta)^b^	TNFα (Phase II)^b^	
1p31	120–130	.72–.80			0.02	0.006	15 Mb from *IL12RB2*
1p21-1q24	130–180	.71–.80	0.01				
2p22-2p16	54–80	.68–.80				0.01	
2q14	120–128	.75–.76		0.003			
2q21-2q24	146–176	.68–.75		**0.0003**			
2q27	250–260	.73–.80		0.03			17 Mb from *SLC11A1*
5p13-5q22	64–114	.69–.77		0.003			
7p22-7p21	0–34	.68–.82	0.002				*IL6*
7q35-7q36	158–172	.51–.57		0.01			
8p12-8q11	64–72	.73–.77		0.02			
8q21-8q23	104–124	.64–.75			0.005	0.02	
9p21-9q12	54–66	.72–.78				0.007	
10q24-10q24	121–163	.57–.81	0.04				
11p15	0–16	.69–.73	0.03				
11q14-11q23	88–118	.72–.84			0.003	0.003	
14p13-14q11	2–24	.51–.77		0.006			
14q21-14q24	55–82	.64–.83		0.02			
19p13-19q12	40–54	.75–.76				0.007	
20q13	64–89	.47–.65	0.02				*MC3R* and *CTSZ*
21q22	38–44	.56–.62			0.0005	0.01	*IFNGR2*
22p13-22q11	2–46	.65–.76	0.007			0.02	

cM = centimorgans, Mb = Megabases.

P-values in boldface indicate results attaining suggestive significance according to Lander-Kruglyak criteria.

a cM range indicates locations significant at the nominal α = 0.05 level.

b The first column of linkage results for the TNFα phenotype are for pooled p-values, and the second column is for the Phase II data analyzed alone.

## Discussion

In this report, we present a full genome scan of a Ugandan population with different responses to Mtb exposure and infection. To our knowledge, this is the largest sample from a TB-endemic population analyzed with a full genome-wide linkage scan. Also, this analysis includes both HIV positive and negative individuals, so these findings are relevant to a general population. This is also the first investigation of a novel phenotype, PTST-, which is an indication of resistance to Mtb infection. We found that two novel regions, on chromosomes 2q21-2q24 and 5p13-5q22, attained suggestive genome-wide significance to PTST-. There may also be a second novel region on chromosome 2q14, which is close enough to 2q21-2q24 that it cannot be distinguished using sib-pair linkage analysis [28]. Since these regions have not been linked to TB in previous studies, future studies will focus on fine mapping these regions. We have also replicated the recent linkage findings of Cooke et al. [8], thus providing the first replication report of the newly published TB candidate genes, *CTSZ* and *MC3R*. By examining distinct phenotypes that represent different stages of the natural history of Mtb infection, we demonstrate that these different stages (i.e. exposure, infection, disease) have distinct genetic influences that underlie the likely different biological mechanisms involved in resistance to infection vs. controlling latent infection vs. progression to active pulmonary TB.

This report provides the first investigation of individuals who have persistently negative TSTs over time despite prolonged and persistent exposure to infectious individuals with TB. Previous studies have suggested that host genetics may play a role in resistance to Mtb infection [11], [12], [14] but did not link this phenotype with any underlying genotype. This study of resistance to Mtb infection therefore provides insight into an early stage of the natural history of disease progression. Since our study is the first to focus on this end of the spectrum of the natural history of Mtb exposure and infection, it is not surprising that we have identified linkage findings that are new to the TB literature. It is very likely that host genetic and immunologic responses differ between resistance to infection, containment of initial Mtb infection and progression of latent Mtb infection to active pulmonary TB. All genetic epidemiological studies of TB to date have focused entirely on the genetic influences underlying the development of disease. Furthermore, since our study observed households over a period of two years, we were uniquely poised to assess the PTST- phenotype. Though it is possible that individuals may convert their skin test beyond this period of observation, it is very unlikely; in our study we observed that most adult TST negative household contacts converted their skin tests within 3 months of study enrollment (∼67%, unpublished data). We observed linkage between this phenotype and several chromosomal regions, some of which were novel, while others – like *SLC11A1* – have been studied extensively. Though the role of SLC11A1 in murine models of TB is well-established, studies of human TB have revealed inconsistent results, and thus its role in the disease phenotype remains controversial [29]. Since we observed linkage between this gene region and PTST-, and not TB, our results may provide a further clue into the role of this gene in the natural history of Mtb infection.

In addition, we observed linkage at the α = 0.05 level to several regions containing potential TB candidate genes. Some of these qualify as replications of previous genetic associations using genome-wide criteria [27]. We detected linkage to a region containing the *IL6* gene. IL6 is known to have both pro- and anti-inflammatory properties in TB [31] and *IL6* knock-out mice succumb to Mtb infection [32]. One case-control study did not find an association between *IL6* and TB [33]; our study is the first to suggest an association. We also observed linkage to the region containing the IL1 complex of genes, with TB as the phenotype; *IL1B* has been associated with TB in Japanese [34], Gambian [35] and Columbian [36] study populations, while *IL1RA* has also been associated with TB in a Gambian population [37]; both genes were associated with pleural TB in a Gujarati Hindu population in England [38]. Additionally, we observed linkage to regions containing genes within the interferon-γ (IFNγ)/interleukin (IL)-12 pathway, including *IL12RB2*, *IL12A* and *IFNGR2*. Though deficiencies in *IFNGR2* have been associated with disseminated non-tuberculous mycobacterial disease [39], [40], this gene was not associated specifically with TB in a case-control study [41]. In the present analysis, *IFNGR2* was linked to TNFα expression levels, which may be the result of crosstalk between the IFNγ and TNFα pathways [42]. *IL12RB2* has been associated with leprosy [43] but not TB and, to our knowledge, *IL12A* has not been associated with TB in any study. Previous genetic epidemiological studies have found associations between *IL12B*, *IL12RB1* and TB, though not with *IL12A* and *IL12RB2* [44]. A possible explanation for this apparent discrepancy could be that risk alleles in *IL12A* and *IL12RB2* are rare and better detectable by linkage [45]. In our analysis, *IL12RB2* was linked to TNFα expression, which may reflect the feedback loop between IFNγ, TNFα, and IL12 [46]. Finally, we observed linkage between all three traits and the chromosome 8 region found to be linked to TB in a whole genome scan in a Moroccan population [30]; our results suggest that this region may contain multiple genes each linked to the different stages of the natural history of Mtb infection and disease, or one gene with pleiotropic effects.

A previous genome scan by Wheeler et al. [23] examined linkage to TB immune phenotypes; these phenotypes were immunoglobulin and IFNγ responses to various Mtb proteins. We found some commonality between their results and ours. The study by Wheeler and colleagues observed linkage to IgG responses to purified protein derivative (PPD) on chromosome 3q23 (p = 0.008) and also linkage to IFNγ responses to PPD, both on chromosome 1q24 (p = 0.0009) and chromosome 19q12 (p = 0.007). In our analysis, the chromosome 1q24 and 3q23 regions were linked to TB and chromosome 19q12 was linked to TNFα responses. This further illustrates the role of these chromosomal regions in immunity related to TB and warrants further investigation.

Though our previous candidate gene analysis [16] demonstrated that some genes were linked to both TNFα and TB, very few regions were linked to multiple traits in this genome scan. There may be several reasons for this. First, the candidate genes in our previous work were chosen specifically because they were part of the TNFα pathway and previously associated with TB. Second, the markers in that study were more densely spaced so that those specific genes were being targeted. And finally, linkage for one trait may not have the same statistical power as for another. Conversely, the present agnostic analysis did not target genes in any specific pathway. Interestingly, several of these candidate genes that were previously only associated with TB were linked with TNFα in the present study, including *IL12RB2*, *NRAMP1 (SLC11A1)*, *IFNGR2*, and the region on chromosome 8 identified by El Baghdadi et al. [30]. This linkage between TB genes and TNFα lends further support to our intermediate phenotype model. These results further support that progression of Mtb infection to disease is a complex trait with likely several underlying distinct genetic risk factors. This complexity of Mtb infection and disease likely explains the inconsistency of results across genetic epidemiological studies. In addition, these results imply that the various stages of the natural history of Mtb infection [9] have unique genetic influences. These results confirm not only that genetic factors influence the interaction between humans and Mtb but more importantly that they differ according to the outcome of that interaction: exposure but no infection vs. infection but no progression to disease vs. progression of infection to disease.

Because of missing genotype and phenotype data, this analysis was limited to 803 individuals, reducing its statistical power. Furthermore, since TNFα was assayed differently in Phase I and II of the study, the data could not be pooled, further affecting power. However, because our results are consistent with current TB immunological models, we think they are unlikely to be false positive findings. This study might have been underpowered to detect genes with smaller effects, since very large sample sizes are required to detect small effects using linkage analysis [45]. Another limitation of the analysis was that empirical p-values could not be computed because we analyzed both full and half sibling pairs together and then defining the appropriate permutation distribution becomes problematic. Future studies will focus on replication in independent populations using an association strategy. Additional studies will use focused microarray and immunological approaches to examine the functional implications of these and other genes.

## Materials and Methods

### Study design

Families were enrolled through the Household Contact Study [26] in Kampala, Uganda. The first phase of the study enrolled households from 1995 through 1999 ascertained through index TB patients who presented to the National TB and Leprosy Programme (NTLP), and had positive acid-fast smear (AFB positive) and positive Mtb cultures; the household was enrolled if the index case lived with at least one person and the individuals in the household as well as the index case provided informed consent to participate in the study. The second phase of the household contact study started enrolling patients from 2002 and continues to the present. Written informed consent was obtained from all individuals in both study phases (or in the case of children, the child provided assent and his/her legal guardian provided written consent on their behalf). Ascertainment in Phase II differed in that index cases were required to be only culture positive for Mtb and, in addition to referral from the NTLP, came to the clinic directly after learning about the study through community sensitization (education) efforts and word-of-mouth. The index case and household members were observed over a two year period in order to capture clinical outcomes as described below. The present analysis of Phase II includes families enrolled from April 2002 through February 2004. Any individuals who withdrew consent were excluded from the analysis. The institutional review boards at University Hospitals of Cleveland and the Uganda Council for Science and Technology approved the study.

### Clinical characterization and phenotypes

After enrollment in the study, all household members were given a full clinical examination, including testing for HIV. Individuals who were suspected to have pulmonary TB received a chest x-ray, and provided sputum samples for culture and AFB smear, or gastric aspirates in the case of young children. Diagnosis of TB disease in contacts was based on ATS criteria [10]; all individuals diagnosed with active TB disease received standard TB therapy for 6 months. In this analysis, “TB case” refers to all individuals with culture-confirmed TB [10]; this includes the index case, co-prevalent cases (diagnosed within 3 months of enrollment), and incident cases (development of TB more than 3 months after baseline study assessment).

Tuberculin skin testing (TST) was done for all individuals in the home, using purified protein derivative (PPD) with the Mantoux method. A positive TST was defined as an induration of 5 mm or greater in children less than or equal to 5 years of age or in HIV positive individuals, or an induration of 10 mm or greater in HIV negative individuals aged greater than 5 years. Skin tests were repeated 3, 6, 12, and 24 months after enrollment in individuals who were TST negative at baseline assessment. A persistently negative TST (PTST-) was defined as a negative skin test on two or more occasions over 2 years of observation in an individual who had been living with the index case. By our definition, these individuals did not go on to develop TB. Individuals who had a negative TST at the time of study enrollment but converted to a positive TST during study follow-up were included in the LTBI group for this analysis. In Phase II of the household contact study, all individuals with positive TST results were offered Isoniazid preventative therapy.

In addition to these clinical outcomes, we also examined cytokine production by whole blood stimulation with soluble Mtb ligands enriched for molecules secreted by Mtb (culture filtrate). Whole blood was stimulated with culture filtrate (10 µg/ml), incubated for 24 hours, and TNFα measured by ELISA in culture supernatant. Additional details regarding the methods for these assays can be found elsewhere [15], [17]. This study measured TNFα expression in response to Mtb culture filtrate proteins and glycolipids.

### Data analysis

Prior to further analysis, TNFα expression variables were adjusted for demographic differences between study cohorts [15]. To examine differences in TNFα between individuals PTST-, with latent Mtb infection (LTBI), and TB, we used nonparametric methods since TNFα values were still not normally distributed even after covariate adjustment and log transformation. This analysis was restricted to HIV negative individuals only so that results would not be confounded by immunosuppresion. Since the ELISA kit and source of culture filtrate differed between Phase I and II of the study [15], we conducted linkage analysis for the TNFα variable separately by study cohort and then combined p-values using Fisher's method [47]; we present the pooled results and results for Phase II alone since the latter data were more complete [16]. Since our diagnosis of TB and PTST- did not differ by study phase, we combined the cohorts for those analyses.

### Molecular methods

DNA was obtained from buffy coat specimens extracted from blood samples taken as part of the study; further detail about DNA preparation is described elsewhere [16]. A microsatellite genome scan was conducted by the Mammalian Genotyping Service at the Center for Medical Genetics in Marshfield, Wisconsin. Four hundred microsatellite markers (Screening Set 16), with an average intermarker spacing of 9.04 cM, were genotyped across the 22 autosomes, chromosome X, and chromosome Y for 828 individuals. The average marker heterozygosity was 0.777 (range 0.462–0.941).

There were several steps involved in genotype cleaning. Eleven of the 400 markers were removed from the analysis because they were in significant Hardy-Weinberg disequilibrium among the founders (p<0.0001), using the method of Montoya-Delgado et al. [48]. We genotyped 18 subjects in duplicate in order to estimate a genotype error rate. The proportion of discordant genotypes in the replicate samples was 0.417%, across all 400 markers. No marker showed large discrepancies compared with any other and no individual sample showed higher error rates compared with any other. Discordant genotypes were deleted prior to further analysis. In addition, Marshfield blindly genotyped DNA samples for estimating typing error, and arrived at an estimated 0.63% rate, most of which were 1 base shifts in allele size. With the genotype data itself, Marshfield provided a number of reports, some of which flagged alleles that were unexpected from the assay (e.g. more or less repeats than had been seen in previous study populations, putative “null” alleles). Alleles at four of these markers segregated within families, so they were retained in the analysis, but the remainder were treated as genotype errors and deleted from the analysis.

Then, we validated pedigree structures using RELTEST [49] and RELPAIR [50] (Figure 1). When these methods identified relationships inconsistent with the molecular data, we queried the on-site staff in Uganda to clarify these relationships. If the molecular data were still inconsistent with the reported relationship, the individual in question was removed from the analysis (N = 13). We also confirmed gender as reported in the database with X and Y chromosome genotypes, and discrepant results were queried and corrected according to the patients' clinic charts; however, some discrepancies were unresolvable for these individuals and their genotypes were deleted from the analysis (N = 12) since DNA contamination or poor quality was suspected. Finally, Mendelian inconsistencies were detected using MARKERINFO (S.A.G.E. v5.3), and inconsistent genotypes were deleted from the data using PedScrubber (courtesy of Dr. Robert Igo, Jr.). In total, 13.6% of the genotypes (including Mendelian errors and poor quality genotypes) were coded as missing. Marker map locations were based on the Screening Set 16 map. Because chromosome 8 inversions have been reported in some populations [51], we verified our chromosome 8 marker order by conducting two-point marker-to-marker linkage analysis using LODLINK (S.A.G.E. v5.3); this analysis demonstrated that the published marker order was correct (data not shown).

### Statistical genetic analysis

Prior to further analysis, we examined the autosomal marker data for population substructure, since the households were enrolled in two different time periods with slightly different ascertainment criteria. We used the STRUCTURE program [52] to attempt to cluster together families with similar subpopulation allele frequencies. This approach has been used successfully in a family study of African Americans to partition families into subpopulations [53]. We attempted 2, 3, and 4 subpopulations, but families did not fall into clusters. Since this analysis supports genetic homogeneity within our study population, we analyzed the data together.

Multipoint analysis of the autosomes and X chromosome was conducted using a model-free approach. Marker allele frequencies in the founders were estimated using FREQ (S.A.G.E. V5.3), and the proportion of alleles shared identical by descent by each relative pair was estimated using GENIBD (S.A.G.E. v5.3). Linkage analysis was conducted using the Haseman-Elston method [54], [55], which regresses a measure of sibpair trait similarity on the proportion of alleles shared identical by descent. Though a quantitative trait linkage method, this model is also applicable for binary traits such as TB and PTST-. This method, as implemented in SIBPAL (S.A.G.E. v5.3), has been recently extended to also incorporate half sibling pairs [56]. Originally, the measure of sibling pair trait similarity was parameterized as the trait difference squared [55]; in recent years, this method was extended to model the dependent trait as the mean-corrected sibpair trait sum [54], and a weighted combination of these two [57], the latter method being referred to as “W4” within SIBPAL. Since trait variance, heritability, and ascertainment method all have an impact on the “best” parameterization of the dependent trait [58], we analyzed the three phenotypes using the original difference squared and W4 options; in all cases, the binary trait analyses were most significant using the difference squared option because of the relatively small number of concordantly affected pairs, while the results for TNFα were most significant using the W4 option. We also included HIV status as a covariate, parameterized as the sibpair sum, in all Haseman-Elston regression models to account for variability due to HIV seropositivity. Genome-wide statistical significance was determined using standard criteria [27]; extent of linked region is reported for markers significant at α = 0.05. Information content within linked regions was evaluated using the method proposed by Krugylak et al. [59], as implemented in MLOD (S.A.G.E. v5.3).
